# Rapidly progressive subungual gray-brown melanonychia

**DOI:** 10.1016/j.jdcr.2024.01.013

**Published:** 2024-01-28

**Authors:** Caroline Echeandia-Francis, Michael R. Nock, Peggy Myung, Amanda E. Zubek

**Affiliations:** a Yale School of Medicine, New Haven, Connecticut; b Department of Dermatology, Yale School of Medicine, New Haven, Connecticut; c Department of Pathology, Yale School of Medicine, New Haven, Connecticut

**Keywords:** digital mucous cyst, digital myxoid pseudocyst, melanonychia, onychodystrophy, subungual lesion

## Case presentation

**Fig 1:**
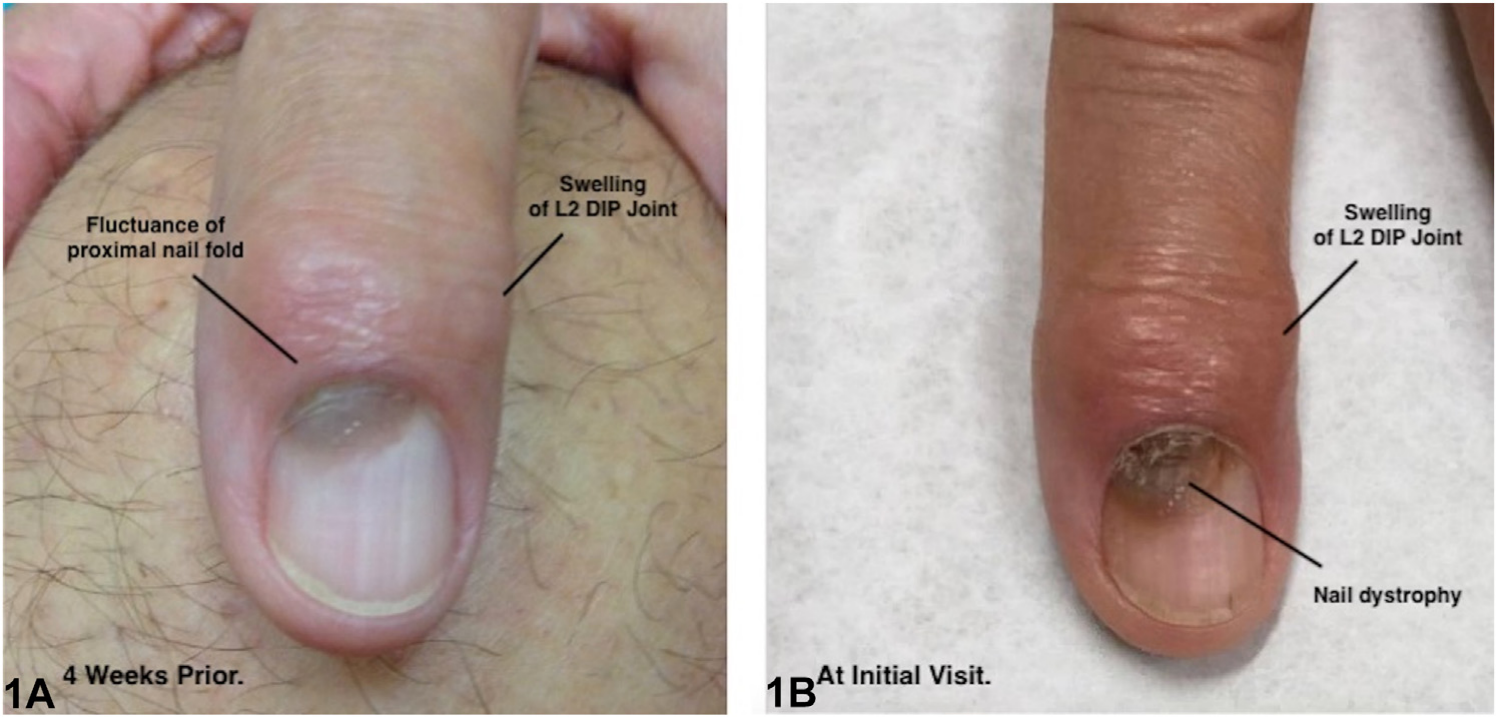

**Fig 2:**
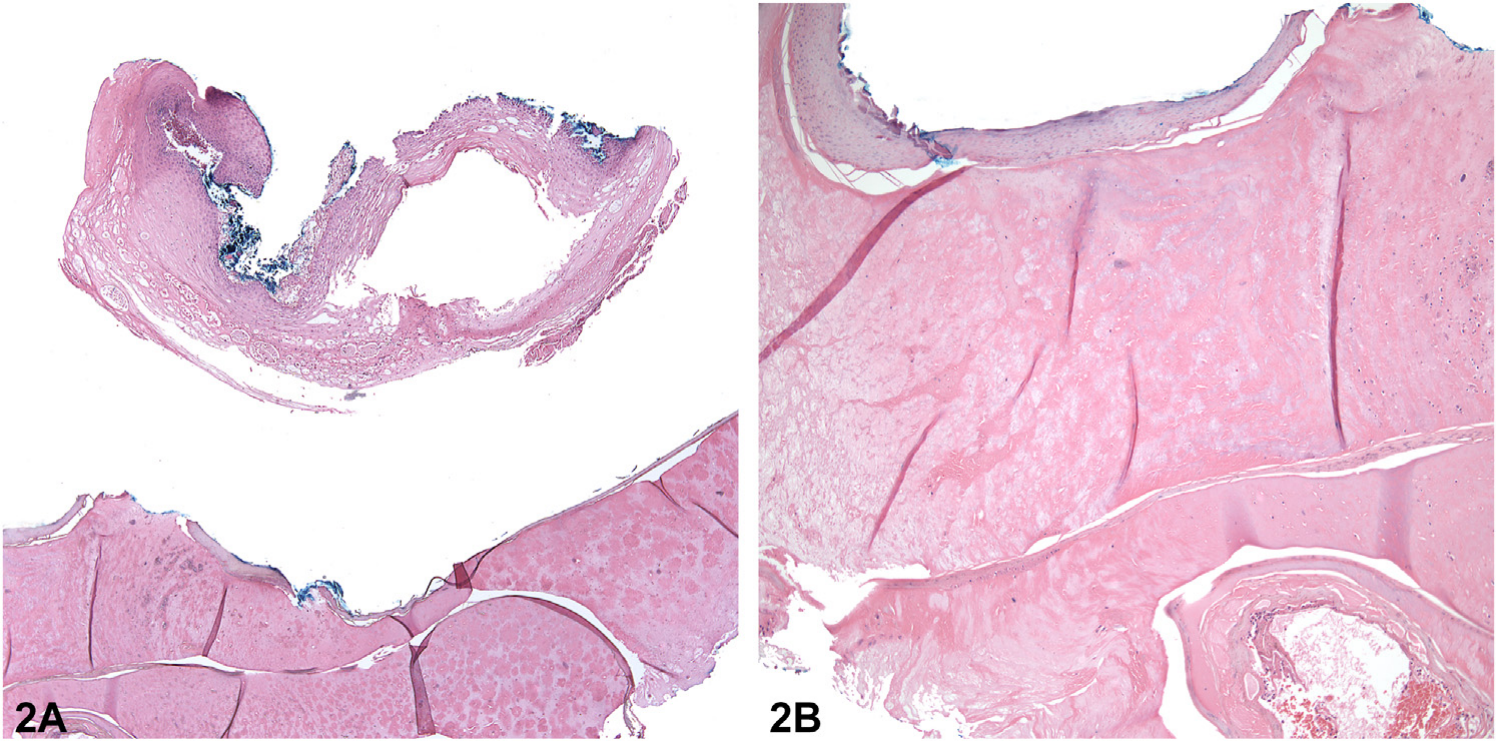

A 73-year-old man presented with a gray-brown proximal subungual lesion on the left second finger (L2) of 3-month duration. In the month prior to presentation, the lesion diameter had doubled (Fig 1, *A* and *B*) and developed overlying nail plate pitting, hyperkeratosis, and irregular dystrophy. The hyponychium was normal, but there was slight edema, erythema, and fluctuance of the proximal nailfold. The nail was asymptomatic; all other fingernails appeared normal. Heberden’s nodes in the distal interphalangeal joints of the bilateral second fingers were observed. The patient denied antecedent trauma to the nail. A nail unit biopsy was performed (Fig 2).

**Question 1: What is the most likely diagnosis?**

A. Nondermatophyte mold onychomycosis
B. Superficial acral fibromyxoma
C. Glomus tumor
D. Digital myxoid pseudocyst
E. Giant cell tumor of the tendon sheath

**Answers:**

**A.** Nondermatophyte mold onychomycosis – Incorrect. Nondermatophyte mold onychomycosis, caused by opportunistic fungi, more commonly presents on the toenails.^1^ Microscopic examination and culture are typically needed for diagnosis and identification of the causative pathogen. On histology, hematoxylin-eosin staining will show hyperkeratosis; hyphae can be more readily identified using periodic acid-Schiff or Grocott methenamine silver staining.^1^
**B.** Superficial acral fibromyxoma – Incorrect. Superficial acral fibromyxoma typically presents as a flesh-colored periungual or subungual nodule and is found to be a nonencapsulated tumor of the dermis on biopsy.^2^
**C.** Glomus tumor – Incorrect. Glomus tumors are benign tumors arising from the glomus body. Subungual glomus tumors are common, often manifest as subungual erythronychia or bluish subungual macules. They are exquisitely painful with pinpoint pressure or cold exposure. Biopsy shows a dermal nodular proliferation of homogenous hyperchromatic glomus cells lining thin-walled vessels.
**D.** Digital myxoid pseudocyst (DMC) – Correct. DMCs are round translucent or skin-colored papules located on dorsolateral fingers or toes. Given the absence of mucoid discharge from subungual DMCs and their location, subungual DMCs are significantly more challenging to diagnose than superficial DMCs. Histology of the lesion revealed mucinous degeneration of the superficial dermis and amorphous collections of pink-bluish material (Fig 2), supporting the diagnosis of a benign subungual DMC.
**E.** Giant cell tumor of the tendon sheath – Incorrect. Giant cell tumor of the tendon sheath are painless slow growing nodules arising from the flexor or extensor tendons of the hands or feet. Biopsy shows deep lobules of epithelioid cells with admixed multinucleate giant cells surrounded by a fibrous pseudocapsule.

**Question 2: Given the rapidly progressive nature of this patient’s lesion, what diagnostic technique would be most efficient and cost-effective?**

A. Magnetic resonance imaging (MRI)
B. Ultrasound
C. Transillumination
D. Aspiration
E. Biopsy of nail unit

**Answers:**

**A.** Magnetic resonance imaging (MRI) – Incorrect. Although MRI has utility in detecting clinically challenging subungual DMCs,^3^ it is costly and may not be ideal for a rapidly progressive lesion for logistical reasons. MRI may detect cyst-joint capsule communication and associated osteoarthritis. Small osteophytes can be more difficult to detect with MRI than other modalities.
**B.** Ultrasound – Incorrect. Although ultrasound is faster and more cost effective for diagnosing subungual DMCs than MRI with higher sensitivity for small lesions, this modality requires access to practitioners experienced in nail unit ultrasonography and its sensitivity is operator dependent.
**C.** Transillumination – Incorrect. Transillumination can be a helpful ancillary test in making the clinical diagnosis of a subungual DMC, especially since it is noninvasive and efficient.^4^ However, transillumination cannot differentiate between different types of cysts (eg, ganglion vs mucinous pseudocyst) and therefore, cannot provide definitive diagnosis.
**D.** Aspiration – Incorrect. While aspiration of the lesion may have revealed mucinous contents characteristic of DMCs, aspiration would be more challenging and painful for a DMC in a subungual location. A lack of mucin in the aspirate would not rule out the diagnosis nor provide an alternative diagnosis. This technique would be appropriate for a DMC that is overlying the nail matrix and therefore easily accessible.
**E.** Biopsy of nail unit – Correct. Although a more invasive technique, biopsy of the nail unit allows for definitive histopathologic diagnosis and is readily available with rapid turnaround time. Quick, definitive diagnosis should be the priority for a rapidly progressing subungual pigmented lesion. Risk of infection and permanent nail dystrophy must be carefully considered. Nonetheless, all the modalities mentioned above have specific and individualized utility and may be indicated depending on the clinical scenario. Some may be more useful than others or used in combination for surgical management planning.

**Question 3: What is the most effective definitive treatment for this symptomatic lesion?**

A. Observation
B. Surgical excision
C. Puncture incision and drainage
D. Intralesional steroid injection
E. Sclerosant injection

**Answers:**

**A.** Observation – Incorrect. Although DMCs are benign and do not require treatment, they are usually not self-resolving. Unless the lesion is symptomatic, observation is often the preferred approach to management.
**B.** Surgical excision – Correct. Surgical excision of the lesion should be considered first-line treatment if the DMC is symptomatic. While surgical excision has potential complications, including infection and nail deformity, this approach has the highest cure rate among treatment options (mean: 95%).^5^ Surgical removal of any associated osteophytes and partial capsulectomy can increase the cure rate^5^; alternatively, surgical excision followed by partial grafting with the cyst’s overlying skin is a more conservative yet effective surgical intervention.^5^
**C.** Puncture incision and drainage – Incorrect. Simple puncture incision and drainage should be avoided given the high likelihood of DMC recurrence following the procedure and since the procedure may expose a patient to an increased risk of infection.
**D.** Intralesional steroid injection – Incorrect. Given that intralesional steroid injection has a relatively lower mean cure rate (61%)^5^ and an associated risk of skin atrophy and bone resorption, intralesional steroid injection should only be considered after surgical excision and sclerosant injection for DMCs.^5^
**E.** Sclerosant injection – Incorrect. Sclerosant injection is an important option to consider for symptomatic DMCs if surgical excision is contraindicated in a patient. Sclerosant injection has an estimated mean cure rate of 77% for DMCs.^5^

## Conflicts of interest

None disclosed.
